# Stiffness evaluation of continuum robots based on the energy method and castigliano’s second theorem

**DOI:** 10.3389/frobt.2025.1523619

**Published:** 2025-03-25

**Authors:** Mengxue Yang, Zhicheng An, Zechen Lin, Yuhang Wang, Tongtao Pang, Fuxin Du

**Affiliations:** ^1^ Beijing Tian Tan Hospital, Capital Medical University, Beijing, China; ^2^ School of Mechanical Engineering, Shandong University, Jinan, China; ^3^ Shandong Institute of Medical Device and Pharmaceutical Packaging Inspection, NMPA Key Laboratory for Quality Evaluation of Medical Materials and Biological Protective Devices, Jinan, China; ^4^ Qilu Hospital of Shandong University Dezhou Hospital, Dezhou, China; ^5^ Key Laboratory of High-Efficiency and Clean Mechanical Manufacture of MOE, Jinan, China

**Keywords:** neurosurgery, notched continuum robots, stiffness, energy method, castigliano’s second theorem

## Abstract

**Introduction:**

Continuum robots are studied and applied in neurosurgery due to their high flexibility and adaptability. The basic performance of continuum is mainly evaluated by stiffness, but there is no systematic and universal evaluation system.

**Methods:**

In this paper, a general experimental platform for continuum robots is designed, based on which the fundamental performance of the notched continuum robot used in neurosurgery is evaluated. The continuum stiffness evaluation method based on energy method and Castigliano’s second theorem is proposed. By solving the internal force and energy of the notched continuum in sections, the stiffness model of single-segment and double-segment series continuum is established. The relationship between the stiffness of the continuum and the bending angle is obtained.

**Results:**

The simulation and experimental results show that under the condition of small deformation angle, the spatial stiffness model obtained by strain energy basically conforms to the actual model, which verifies the correctness and rationality of the stiffness calculation method proposed in this paper.

**Discussion:**

This paper is of significant importance to promote the performance evaluation and optimization of continuum.

## 1 Introduction

Continuum robots are characterized by elastic structures and infinite degrees of freedom, lacking discrete joints and rigid links typical of traditional rigid robots (Webster III and Jones, 2010). They exhibit a high degree of dexterity not found in traditional robots, coupled with strong adaptability to workspace constraints. Consequently, they find extensive applications in specialized fields such as medical devices, search and rescue, demonstrating exceptional performance (He et al., 2018). Continuum robots have shown great potential in neurosurgery, such as cerebral hemorrhage aspiration (Burgner et al., 2013), transnasal skull base tumor resection (Zhang et al., 2022), and other operations. Currently, continuum robot designs include hinge-based, segmented, concentric tube, and notched configurations among others (Li and Du, 2013).

Hinge-based continuum robots connect their joints through ball sockets or hinges. Professor Zheng Li from The Chinese University of Hong Kong has developed corresponding kinematic and workspace models based on the assumption that joint curvatures along the continuum bending curve are equal (Li et al., 2016). While these robots exhibit out-standing flexibility, friction between ball sockets can cause noticeable hysteresis and uneven bending during motion. Segmented continuum robots, powered by drive cables with central elastic rods providing compensatory force, approximate constant curvature behavior during bending but may occlude the central channel. In 2006, researchers in-cluding Nabil Simaan from Columbia University and Kai Xu from Shanghai Jiao Tong University proposed using Nitinol alloy tubes instead of drive cables to increase robot stiffness (Xu and Simaan, 2006; Xu and Simaan, 2008; Simaan et al., 2009). Similarly, researchers such as Bin He from Tongji University utilized superelastic Nitinol alloys to design a three-backbone continuum robot, establishing kinematic and dynamic models (He et al., 2013). Concentric tube continuum robots, introduced by Robert James Webster III from Vanderbilt University, achieve bending motion through pre-curved elastic sheaths and inner tube feeding, applied in medical fields for complex surgical operations. However, inherent elastic forces between the inner and outer tubes contribute to significant errors in the robot’s kinematic model, challenging precise end-effector control (Webster et al., 2008). Notched continuum robots, initially proposed by Johns Hopkins University (Badescu and Mavroidis, 2004), use linear drive mechanisms to form joints at incisions, compensating for uneven bending in the continuum, providing improved stiffness. Researchers from Harbin Institute of Technology (Wilkening et al., 2017; Gao et al., 2016a) investigated triangular and square notched continuum robots, establishing their mechanical and kinematic models. Zhang et al. (2024b), Zhang et al. (2024a) propose to mix the rectangular cut continuum with the concentric tube continuum and establish the kinematic models.

In current research, the focus on continuum robots primarily centers around structural innovations, precise control, and performance evaluation. Due to their unique operating environment, continuum robots are required not only high flexibility but also a certain degree of rigidity, as they must balance their load capacity and motion precision while ensuring adequate adaptability and safety (Gu and Ren, 2023; Lin et al., 2024).

Stiffness analysis is a crucial aspect of the design and control of continuum robots, determining the relationship between deformation and forces in these systems. Numerous experts have conducted in-depth studies on stiffness metrics. Selig and Ding utilized screw theory (Dai, 2012) to analyze the flexibility and stiffness matrices of beams. Pei et al. (2009a) investigated the flexibility of wheel flexible joints. Ding and Dai (Pei et al., 2009b) explored the spatial continuum flexibility of serial and parallel mechanisms based on screw theory and Lie group theory, employing eigenvectors and eigenvalues to identify principal screws within mechanisms. Awtar and Sen (2010) proposed a generalized constraint model for analyzing the flexural flexibility and stiffness of 2D beams. Tunay introduced the concept of equivalent bending stiffness. Gao et al. (2016b) developed a mathematical model to predict the load postures of single cross-section continuum robotic arms. Qi et al. (2015) analyzed the flexibility characteristics of a novel planar spring continuum robot. Gravagne et al. (2003) discussed the dynamics of planar continuum backbone sections using a large deflection dynamic model. Trivedi et al. (2007) introduced a novel modeling approach for flexible robotic arms that incorporates material nonlinearity and the effects of distributed weight and payload. Camarillo et al. (2008) proposed a new linear model for converting desired beam configurations to tendon displacements and *vice versa*. Fraś et al. (2014) described the design and implementation of a static model used for position estimation of modular medical robotic arms equipped with fiber optic sensors. Sadati et al. (2017) presented a series solution method for static and Lagrangian dynamic analysis of a new variable curvature Cosserat rod. However, a comprehensive and universally applicable evaluation framework is currently lacking.

This paper designs a universal continuum experimental platform, which can realize the driving of multiple segments of cable-driven continuum of different sizes. Based on the energy method and Castigliano’s second theorem, the stiffness of the notched continuum introduced above is modeled. The contributions of this paper as follows:

• This paper designs a universal continuum platform, based on which the stiffness model of the double-segment notched continuum is verified and the stiffness of the serial continuum under different driving strategies is tested.
• Utilizing energy methods and Castigliano’s second theorem, the internal forces and energies of incision-type continuum bodies are sequentially resolved, establishing stiffness models for both single-section and dual-section series-connected continua. The relationship between continuum stiffness and bending angle is derived.
• Simulation and experimental results indicate that, under small deformation conditions, the spatial stiffness models derived from strain energy align closely with practical models. The stiffness relationships of dual-section series-connected notched under varying numbers of driving cables are established as follows: External 4 + Internal 4 > External 2 + Internal 4 > External 4 + Internal 2 > External 2 + Internal 2.

The remaining parts of this paper are as follows: Section 2 describes the structure of the universal experimental platform for continuum robot. Section 3 establishes stiffness models for single-section and dual-section series-connected notched continuum. Section 4 conducts simulations of the proposed stiffness analysis models and validates their effectiveness through experimentation. Section 5 provides a summary of the entire paper.

## 2 The structure of the universal platform

**FIGURE 1:**
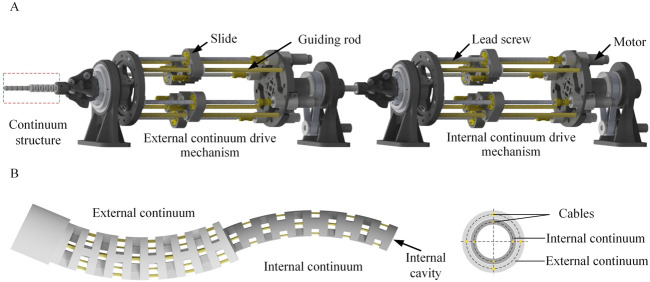
The structure of the universal platform. **(A)** The universal continuum experimental platform. The platform consists of three parts: continuum structure, external continuum drive mechanism and internal continuum drive mechanism. **(B)** The specific structure of the dual-segment notched continuum robot.

To realize the driving and testing of continuum of various sizes and different numbers of drive cables, this paper designs a universal continuum experimental plat-form, as shown in Figure 1A. The overall structure of the continuum experimental platform can be divided into two parts, namely, the main driving mechanism and the linear module. The main driving mechanism consists of 4 sets of lead screw slider mechanisms, a front base, a rear base and a front wire guide mechanism. The pressure sensor is installed on the slide. One end of the pressure sensor is installed together with the slide, and the other end is installed with a V-groove guide wheel to transmit the drive cable and measure the pressure of the drive cables. The structure of the double-segment series continuum is shown in Figure 1B.

The structure of the double-segment series continuum is shown in Figure 1B. The structure of the double-segment series continuum is shown in Figure 1B. The parameters of the external and internal continuum are shown in Table 1.

**TABLE 1:** The parameters of the external and internal continuum.

	External continuum	Internal continuum
Diameter of the drive cable	0.8 mm	0.8 mm
Total length	42 mm	82 mm
Distribution diameter of drive cable	9 mm	4 mm
Height of notches	2 mm	2 mm
Outer diameter	11.2 mm	6.8 mm
Inner diameter	6.9 mm	3 mm
Number of notched	10 mm	20 mm
Length of beam	2 mm	1.9 mm
Width of beam	2 mm	2.8 mm

## 3 Stiffness evaluation in series-connected continuum

This section models the stiffness of the notched continuum based on the energy method (Straughan, 2013) and the Castigliano’s second theorem (Eastwood et al., 2016). Additionally, the relationship between the spatial stiffness of serial continuum and bending angles is obtained. Finally, the stiffness of serial continuum under four different driving strategies are compared.

### 3.1 Energy method and stiffness

When calculating the end displacement of the continuum, this chapter adopts the energy method. Assuming the elastic body has no rigid displacements under the constraints of the supports, and is subjected to *n* external forces, the strain energy stored in the system due to these external forces is denoted as $V_{\varepsilon }$ . If an incremental force $\Delta F_{i}$ is applied to one of these forces $F_{i}$ , the total strain energy of the system becomes $V_{\varepsilon }+\Delta V_{\varepsilon }$ . Altering the sequence of force application involves first applying $\Delta F_{i}$ to the elastic body before applying the external force. Applying $\Delta F_{i}$ results in a displacement $\Delta \left(\Delta F_{i}\right)$ in the direction of this increment. Therefore, the work done by $\Delta F_{i}$ is represented as *W*.

$$V_{\varepsilon }+\;\Delta V_{\varepsilon }=V_{\varepsilon }+\Delta F_{i}\Delta_{i}+\frac{1}{2}\Delta F_{i}\cdot \Delta \left(\Delta_{i}\right)$$ (1)

Neglecting higher-order terms, it can be obtained that

$$\Delta_{i}=\;\frac{\Delta V_{\varepsilon }}{\Delta F_{\text{i}}}$$ (2)

From the Equation 1 and Equation 2, it can be seen that the partial derivative of strain energy with respect to any load $F_{i}$ equals the displacement of the point of action of $F_{i}$ in the direction of $F_{i}$ . This is known as Castigliano’s second theorem, applicable exclusively to linear elastic structures. Additionally, formulas are provided below for the incremental strain energy $\Delta V_{\varepsilon }$ induced by forces of various natures. The incremental strain energy induced by the normal support force $F_{N}$ is shown in Equation 3.

$$\Delta {V_{\varepsilon }}^{F_{N}}=\frac{{F_{N}}^{2}}{2EA}ds$$ (3)

The incremental strain energy induced by the shear force $F_{s}$ is shown in Equation 4.

$$\Delta {V_{\varepsilon }}^{F_{s}}=\frac{k{F_{s}}^{2}}{2GA}ds$$ (4)

The incremental strain energy induced by the bending moment *M* is shown in Equation 5.

$$\Delta {V_{\varepsilon }}^{M}=\frac{M^{2}}{2EI}ds$$ (5)

The incremental strain energy induced by the torque *T* is shown in Equation 6.

$$\Delta {V_{\varepsilon }}^{T}=\frac{T^{2}}{2GI_{P}}ds$$ (6)

where *E* is the modulus of elasticity, *A* is the cross-sectional area of the continuum, *K* is the shear shape coefficient, *I* is the moment of inertia, *G* is the shear modulus of elasticity, $I_{p}$ is the polar moment of inertia.

**FIGURE 2:**
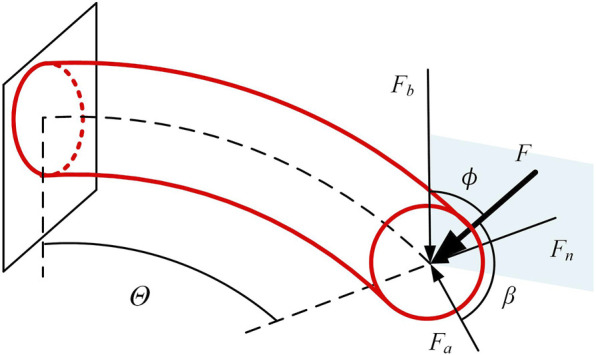
Force conditions at the end of the continuum joint.

Due to the typical bending state of continua in operational conditions, this section is based on the assumption of constant curvature for analyzing the stiffness of continuum under bending. The continuum is typically subjected to three types of end forces: axial force $F_{a}$ , principal normal force $F_{n}$ , and deputy normal force $F_{b}$ , as shown in Figure 2. These forces exert varying effects on continuum, necessitating distinct analytical approaches.

### 3.2 Continuum robot stiffness model

**FIGURE 3:**
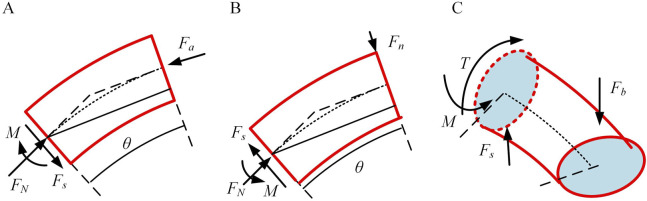
The force on the continuum. **(A)** Axial force situation of continuum element. **(B)** Principal normal force situation of element. **(C)** Tangential normal force situation of element.

The continuum can be envisioned as a smooth circular tube. As shown in Figure 3, consider the overall bending angle Θ of the continuum robot, with a total length *L*. To analyze a small segment of the continuum robot near its end, with a bending angle θ and length *s*. We note the following forces: axial force $F_{a}$ , normal force $F_{N}$ , shear force $F_{s}$ , and bending moment M acting on this segment of the continuum under stress.

As shown in Figure 3A, according to the force balance, the following equation can be obtained:

$$\left\{\begin{array}{c} F_{N}=F_{\text{a}}\cos\theta \;\\ F_{s}=F_{\text{a}}\sin\theta \;\\ M=F_{a}l\left(1-\cos\theta \right)/\Theta \; \end{array}\right.$$ (7)

According to Castigliano’s second theorem, the deformation at the end is related to the strain energy. Let the deformation variable at the end be dx , then it satisfies

$$dx=\frac{\partial \left(\overset{l}{\underset{0}{\int }}\frac{{F_{N}}^{2}}{2EA}ds+\overset{l}{\underset{0}{\int }}\frac{k{F_{s}}^{2}}{2GA}ds+\overset{l}{\underset{0}{\int }}\frac{M^{2}}{2EI}ds\right)}{\partial F_{a}}$$ (8)

Substituting Equation 7 into Equation 8 yields Equation 9:

$$\begin{array}{c} dx=\overset{\Theta }{\underset{0}{\int }}\frac{F_{a}l\cos^{2}\theta }{EA\Theta }d\theta +\overset{\Theta }{\underset{0}{\int }}\frac{kF_{a}l\sin^{2}\theta }{GA\Theta }d\theta +\overset{\Theta }{\underset{0}{\int }}\frac{F_{a}{\left(1-\cos\theta \right)}^{2}}{EI}\frac{l^{3}}{\Theta^{3}}d\theta \\ \;=\frac{F_{a}l}{EA\Theta }\frac{2\Theta +\sin2\Theta }{4}+\frac{kF_{a}l}{GA\Theta }\frac{2\Theta -\sin2\Theta }{4}+\frac{F_{a}}{EI}\frac{l^{3}}{\Theta^{3}}\frac{6\Theta -8\sin\Theta +\sin2\Theta }{4} \end{array}$$ (9)

In the above equation, the first to third terms on the right-hand side represent the deformations at the end caused by axial force, shear force, and bending moment, respectively. *E* and *G* are constants obtainable through experimentation. Axial stiffness is defined as the ratio of axial force to deformation, thus *K_a_* can be described in Equation 10.

$$K_{a}={\left(\frac{l}{EA\Theta }\frac{2\Theta +\sin2\Theta }{4}+\frac{kl}{GA\Theta }\frac{2\Theta -\sin2\Theta }{4}+\frac{1}{EI}\frac{l^{3}}{\Theta^{3}}\frac{6\Theta -8\sin\Theta +\sin2\Theta }{4}\right)}^{-1}$$ (10)

Furthermore, since the continuum is a slender rod with a large length-to-diameter ratio, shear strain is significantly smaller compared to other strain energies. Therefore, it can be neglected, and $K_{a}$ can be simplified to Equation 11.

$$K_{a}={\left(\frac{l}{EA\Theta }\frac{2\Theta +\sin2\Theta }{4}+\frac{1}{EI}\frac{l^{3}}{\Theta^{3}}\frac{6\Theta -8\sin\Theta +\sin2\Theta }{4}\right)}^{-1}$$ (11)

Similarly, as shown in Figure 3B, the following can be derived in Equation 12.

$$K_{n}={\left(\frac{l}{EA\Theta }\frac{2\Theta -\sin2\Theta }{4}+\frac{1}{EI}\frac{l^{3}}{\Theta^{3}}\frac{2\Theta -\sin2\Theta }{4}\right)}^{-1}$$ (12)

When the tangential direction is subjected to an external force $F_{b}$ , not only shear force and bending moment occur, but also torsion is generated, as shown in Figure 3C.

$$T=F_{b}\frac{l}{\Theta }\left(1-\cos\theta \right)$$ (13)

Similarly, the expression of dx and $K_{b}$ are as follows.

$$\left\{\begin{array}{c} dx=\frac{\partial \left(\overset{l}{\underset{0}{\int }}\frac{T^{2}}{2GI_{P}}ds+\overset{l}{\underset{0}{\int }}\frac{k{F_{s}}^{2}}{2GA}ds+\overset{l}{\underset{0}{\int }}\frac{M^{2}}{2EI}ds\right)}{\partial F_{b}}\;\\ K_{b}={\left(\frac{1}{GI_{P}}\frac{l^{3}}{\Theta^{3}}\frac{6\Theta -8\sin\Theta +\sin2\Theta }{4}+\frac{2}{EI}\frac{l^{3}}{\Theta^{3}}\left(\Theta -\sin\Theta \right)\right)}^{-1}\; \end{array}\right.$$ (14)

**FIGURE 4:**
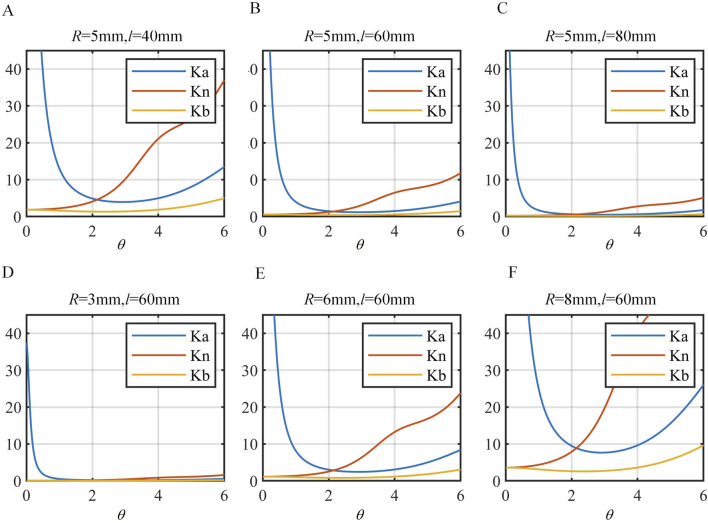
Comparison of stiffness in different directions. **(A)** R=5mm , l=40mm . **(B)** R=5mm , l=60mm . **(C)** R=5mm , l=80mm . **(D)** R=3mm , l=60mm . **(E)** R=3mm , l=60mm . **(F)** R=8mm , l=60mm .

The stiffness of the continuum in three different directions is compared under different lengths *l* and outer diameters *R*, as shown in Figures 4A–F. The distribution of stiffness $K_{a}$ , $K_{n}$ and $K_{b}$ across different orientations shows that within the bending range of (0, 1.5) radians, the stiffness in the principal normal direction significantly exceeds that in the axial and tangential directions. However, at extremely small bending angles, the tangential direction stiffness can surpass the axial direction stiffness.

Analyzing the more generalized spatial stiffness model, as shown in Figure 2, where the continuum subjected to a load *F* at its end experiences a total deformation dx . The force *F* can be decomposed into components $F_{a}$ , $F_{n}$ and $F_{b}$ . Given that the plane containing *F* makes an angle β with the plane defined by directions *a* and *b*, and within that plane, *F* forms an angle φ with direction *b*. Therefore, the magnitudes of the individual force components are shown in Equation 15.

$$\left\{\begin{array}{c} F_{n}=\;F\sin\phi \sin\beta \;\\ F_{\text{a}}=\;F\sin\phi \cos\beta \;\\ F_{b}=\;F\cos\phi \; \end{array}\right.$$ (15)

Similarly, the deformation of *F* at the end can be represented as Equation 16.

$$\vec{dx}=\;\vec{dx^{\text{a}}}+\;\vec{dx^{\text{n}}}+\;\vec{dx^{\text{b}}}$$ (16)

If the loads *F* act individually in three directions, resulting in deformations $dx^{F_{a}}$ , $dx^{F_{n}}$ and $dx^{F_{b}}$ respectively at the ends, the relationship can be expressed as

$$dx=dx^{Fa}\cos^{2}\varphi^{a}+dx^{Fn}\cos^{2}\varphi^{n}+dx^{Fb}\cos^{2}\varphi^{b}$$ (17)

The angles $\varphi_{a}$ , $\varphi_{n}$ and $\varphi_{b}$ represent the angles between the spatial force *F* and the directions of the three component forces. Further derivation yields:

$$\frac{1}{K}=\frac{dx}{F}=\frac{\cos^{2}\varphi^{a}}{K_{a}}+\frac{\cos^{2}\varphi^{n}}{K_{n}}+\frac{\cos^{2}\varphi^{b}}{K_{b}}$$ (18)

### 3.3 Single-section notched continuum stiffness model

**FIGURE 5:**
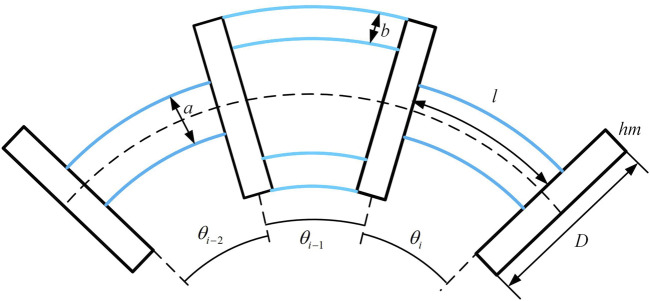
The bending condition of three consecutive joints of the notched continuum robot.

The skeleton diagram of the notched continuum is shown in Figure 5. The constant curvature assumption is adopted to describe the bending of the continuum for computational convenience. The bending angle $\theta_{i}$ at section *i* of the continuum is assumed to be uniform. The total bending angle θ of the continuum satisfies the Equation 19.

$$\Theta =\;\overset{\text{i}}{\underset{0}{\sum }}\theta_{\text{i}}$$ (19)

The cross-section of the continuum consists of two symmetric annular structures. The cross-section is simplified into two symmetrically distributed rectangles. Each rectangle has the length *a*, width *b*, and the distance from the rectangle’s center to the centroid *c*. Therefore, the moments of inertia of the continuum skeleton section about the *x* and *y*-axes, as well as the polar moment of inertia, are given by Equation 20.

$$I_{x}=\frac{ab^{3}}{6},I_{y}=\frac{ba^{3}}{6}+2abc,I_{p1}=\frac{ab^{3}}{6}+\frac{ba^{3}}{6}+2abc$$ (20)

The continuum drive cable studied in this paper is made of nickel-titanium alloy and can be approximated to obey Hooke’s law. Its loading conditions are treated as equivalent to a slender rod. When the drive cable is fully tensioned, friction between the cable and the continuum skeleton is neglected. For computational convenience, this study considers four cables as a unified whole for analysis, with each cable having a cross-section of a uniformly distributed circle.

Therefore, the moments of inertia of the drive wires around the *x/y*-axis and the equivalent polar moment of inertia are shown in Equation 21.

$$I_{2}=\frac{\pi d^{4}}{16}+\frac{\pi d^{2}e^{2}}{2},I_{p2}=\frac{\pi d^{4}}{8}+\pi d^{2}e^{2}$$ (21)

**FIGURE 6:**
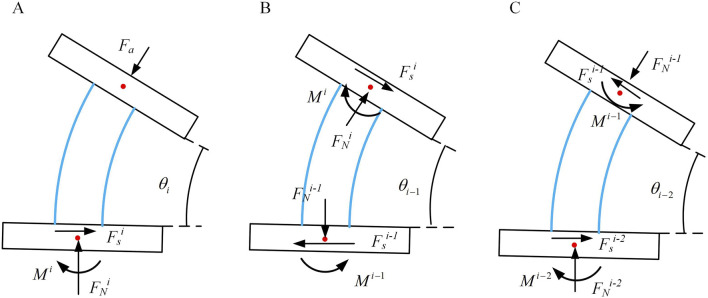
Three consecutive joint bending situations. **(A)** Bending condition of joint *i*. **(B)** Bending condition of joint *i*-1. **(C)** Bending condition of joint *i*-2.

To calculate the axial stiffness, strain energy possessed by the continuum should be determined. The upper and lower annular discs of a joint can be regarded as rigid body, hence they possess no strain energy. The strain energy of the joint is concentrated in the elastic rod, as shown in Figure 6, which shows a schematic of joint *i* near the end of the continuum. $F_{a}$ represents the axial load.

Similar to axial stiffness, the force equilibrium within the internal elemental units of the continuum can be inferred from the previous section. The force distribution at the base of each unit resembles that of the principal normal stiffness. Hence, the strain energy within this joint is shown in Equation 22.

$${V_{\varepsilon }}^{\text{i}}=\overset{l}{\underset{0}{\int }}\frac{{\left({F_{dN}}^{\text{i}}\right)}^{2}}{2E_{1}A}ds+\overset{l}{\underset{0}{\int }}\frac{k{\left({F_{ds}}^{\text{i}}\right)}^{2}}{2G_{1}A}ds+\overset{l}{\underset{0}{\int }}\frac{{\left({M_{d}}^{\text{i}}\right)}^{2}}{2E_{1}I_{\text{x}}}ds$$ (22)

Considering the case of the *i-1* joint. As shown in Figure 6A, based on its geometric features, the equations of static equilibrium can be formulated in Equation 23.

$$\left\{\begin{array}{c} {F_{s}}^{\text{i }-\;1}={F_{s}}^{\text{i}}\cos\theta +{F_{N}}^{\text{i}}\sin\theta \;\\ {F_{N}}^{\text{i }-\;1}={F_{s}}^{\text{i}}\sin\theta -{F_{N}}^{\text{i}}\cos\theta \;\\ M^{\text{i}}-M^{\text{i }-\;1}={F_{s}}^{i-1}l\sin\theta /\theta_{\text{i }-\;1}+{F_{N}}^{i-1}l\left(1-\cos\theta \right)/\theta_{\text{i }-\;1}\; \end{array}\right.$$ (23)

The strain energy within the *i-1* joint shown in Equation 24.

$${V_{\varepsilon }}^{\text{i }-\;1}=\overset{l}{\underset{0}{\int }}\frac{{\left({F_{dN}}^{\text{i }-\;1}\right)}^{2}}{2E_{1}A}ds+\overset{l}{\underset{0}{\int }}\frac{k{\left({F_{ds}}^{\text{i }-\;1}\right)}^{2}}{2G_{1}A}ds+\overset{l}{\underset{0}{\int }}\frac{{\left({M_{d}}^{\text{i }-\;1}\right)}^{2}}{2E_{1}I_{y}}ds$$ (24)

The strain energy within the *i-2* joint is computed similarly to that within the *i-1* joint. Following this method, for any joint *n* (where *n* = 1, 2 … *i*), the total strain energy of the continuum skeleton can be expressed, then the total strain energy of the continuum skeleton can be expressed as Equation 25.

$$V_{\varepsilon }=\overset{i}{\underset{n=1}{\sum }}{V_{\varepsilon }}^{n}$$ (25)

To express the axial stiffness of the notched continuum further in Equation 26.

$$\left\{\begin{array}{c} dx=\frac{\partial \left(V_{\varepsilon }\right)}{\partial F_{a}}\;\\ K_{a}=\frac{F_{a}}{dx}\; \end{array}\right.$$ (26)

**FIGURE 7:**
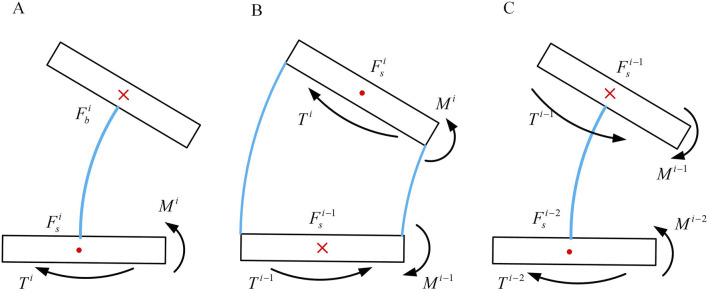
Three consecutive joint bending situations. **(A)** Bending condition of joint *i*. **(B)** Bending condition of joint *i*-1. **(C)** Bending condition of joint *i*-2.

To determine the principal normal stiffness, considering the *i-1*st joint scenario, the input force at this joint is influenced by the end force at the first joint. As shown in Figure 7, based on geometric characteristics, the static equilibrium equations can be derived.

$$\left\{\begin{array}{c} {F_{s}}^{\text{i }-\;1}={F_{s}}^{\text{i}}\cos\theta -{F_{N}}^{\text{i}}\sin\theta \;\\ {F_{N}}^{\text{i }-\;1}={F_{s}}^{\text{i}}\sin\theta +{F_{N}}^{\text{i}}\cos\theta \;\\ M^{\text{i}}-M^{\text{i }-\;1}={F_{s}}^{i-1}l\sin\theta /\theta_{\text{i }-\;1}+{F_{N}}^{i-1}l\left(1-\cos\theta \right)/\theta_{\text{i }-\;1}\; \end{array}\right.$$ (27)

Through the above equation, the forces at the bottom of the second joint and subsequently determine the strain energy within the second joint can be solved. The strain energy of the *i-2*nd joint aligns with Equation 27. Similarly, can be derived (*n* = 1, 2 … *i*), thus obtaining the total strain energy of the continuum. This enables the stiffness of the notched continuum in the principal normal direction can be expressed.

To solve for the deputy normal stiffness of the notched continuum, as shown in Figure 7A, when subjected to an external force $F_{b}$ , a torque is generated. The force situation is illustrated in Figure 7C. Similar to the static analysis of the *i-2*nd joint and Equations 13, 14, the strain energy of the continuum joints can be determined. In the *i-1*st joint, the condition satisfied is shown in Equation 28.

$$\left\{\begin{array}{c} {F_{s}}^{i}={F_{s}}^{i-1}\;\\ M^{i-1}=M^{i}-2{F_{s}}^{i}\frac{l}{\theta_{i-1}}\sin\frac{\theta }{2}\;\\ T^{i-1}=T^{i}+{F_{s}}^{i-1}l\left(1-\cos\theta \right)/\theta_{i-1}\; \end{array}\right.$$ (28)

By analogously applying the static equilibrium relationships in the *i-2*nd joint, we can similarly derive (*n* = 1, two … *i*) the total strain energy of the continuum. The stiffness of the notched continuum in the transverse direction can be expressed.

Analyzing the axial stiffness of the drive cable, when the axial load $F_{a}$ is applied to the end of the continuum, as indicated by earlier discussions and considering static equilibrium and deformation coordination relationships, dx and *K_a_* can be shown in Equation 29 and Equation 30.

$$\begin{array}{c} dx=\overset{\Theta }{\underset{0}{\int }}\frac{F_{a}l\cos^{2}\theta }{E_{2}A\Theta }d\theta +\overset{\Theta }{\underset{0}{\int }}\frac{kF_{a}l\sin^{2}\theta }{G_{2}A\Theta }d\theta +\overset{\Theta }{\underset{0}{\int }}\frac{F_{a}{\left(1-\cos\theta \right)}^{2}}{E_{2}I_{2}}\frac{l^{3}}{\Theta^{3}}d\theta \\ \;=\frac{F_{a}l}{E_{2}A\Theta }\frac{2\Theta +\sin2\Theta }{4}+\frac{kF_{a}l}{G_{2}A\Theta }\frac{2\Theta -\sin2\Theta }{4}\\ \;+\frac{F_{a}}{E_{2}I_{2}}\frac{l^{3}}{\Theta^{3}}\frac{6\Theta -8\sin\Theta +\sin2\Theta }{4} \end{array}$$ (29)

$$K_{a}={\left(\frac{l}{E_{2}A\Theta }\frac{2\Theta +\sin2\Theta }{4}+\frac{kl}{G_{2}A\Theta }\frac{2\Theta -\sin2\Theta }{4}+\frac{1}{E_{2}I_{2}}\frac{l^{3}}{\Theta^{3}}\frac{6\Theta -8\sin\Theta +\sin2\Theta }{4}\right)}^{-1}$$ (30)

When analyzing the primary normal stiffness of the driving cable, and considering that the end of the continuum is subjected to a load $F_{n}$ in the primary normal direction, as previously discussed. Thus *dx* and *K_n_* can be described in Equation 31 and Equation 32.

$$\begin{array}{c} dx=\overset{\Theta }{\underset{0}{\int }}\frac{F_{n}l\sin^{2}\theta }{E_{2}A\Theta }d\theta +\overset{\Theta }{\underset{0}{\int }}\frac{kF_{n}l\cos^{2}\theta }{G_{2}A\Theta }d\theta +\overset{\Theta }{\underset{0}{\int }}\frac{F_{n}\sin^{2}\theta }{E_{2}I_{2}}\frac{l^{3}}{\Theta^{3}}d\theta \\ \;=\frac{F_{n}l}{E_{2}A\Theta }\frac{2\Theta -\sin2\Theta }{4}+\frac{kF_{n}l}{G_{2}A\Theta }\frac{2\Theta +\sin2\Theta }{4}+\frac{F_{n}}{E_{2}I_{2}}\frac{l^{3}}{\Theta^{3}}\frac{2\Theta -\sin2\Theta }{4} \end{array}$$ (31)

$$K_{n}={\left(\frac{l}{E_{2}A\Theta }\frac{2\Theta -\sin2\Theta }{4}+\frac{kl}{G_{2}A\Theta }\frac{2\Theta +\sin2\Theta }{4}+\frac{1}{E_{2}I_{2}}\frac{l^{3}}{\Theta^{3}}\frac{2\Theta -\sin2\Theta }{4}\right)}^{-1}$$ (32)

When analyzing the secondary normal stiffness, upon the continuum’s end being subjected to a load $F_{b}$ in the deputy normal direction, resulting in reactions and moments at the end of the cable. Thus *dx* and *K_b_* can be described in Equation 35 and Equation 34.

$$\begin{array}{c} dx=\overset{\Theta }{\underset{0}{\int }}\frac{F_{b}{\left(1-\cos\theta \right)}^{2}}{G_{2}I_{P}}\frac{l^{3}}{\Theta^{3}}d\theta +\overset{\Theta }{\underset{0}{\int }}\frac{kF_{b}l}{G_{2}A\Theta }d\theta +\overset{\Theta }{\underset{0}{\int }}\frac{4F_{b}\sin^{2}\frac{\theta }{2}}{E_{2}I_{2}}\frac{l^{3}}{\Theta^{3}}d\theta \\ \;=\frac{F_{b}}{G_{2}I_{P2}}\frac{l^{3}}{\Theta^{3}}\frac{6\Theta -8\sin\Theta +\sin2\Theta }{4}+\frac{kF_{b}l}{G_{2}A}+\frac{2F_{b}}{E_{2}I_{2}}\frac{l^{3}}{\Theta^{3}}\left(\Theta -\sin\Theta \right) \end{array}$$ (33)

$$K_{b}={\left(\frac{1}{G_{2}I_{P2}}\frac{l^{3}}{\Theta^{3}}\frac{6\Theta -8\sin\Theta +\sin2\Theta }{4}+\frac{kl}{G_{2}A}+\frac{2}{E_{2}I_{2}}\frac{l^{3}}{\Theta^{3}}\left(\Theta -\sin\Theta \right)\right)}^{-1}$$ (34)

Comparing the coupled stiffness between the continuum and the cables in different directions, the expressions for their stiffness along the axial have been derived, principal normal, and deputy normal directions in preceding sections. Thus, the combined stiffness in these three orientations can be determined, as outlined in Hong et al. (2022).

$$\left\{\begin{array}{c} K_{a}=K_{a-C}+K_{a-L}\;\\ K_{n}=K_{n-C}+K_{n-L}\;\\ K_{b}=K_{b-C}+K_{b-L}\; \end{array}\right.$$ (35)

In this context, subscript *C* denotes the continuum, while subscript *L* represents the drive cable. The iterative computations are performed on the directional stiffness of the notched continuum and drive cable coupled model using MATLAB. The parameter definitions and values used for modeling are detailed in Table 1, outside the continuum section.

**FIGURE 8:**
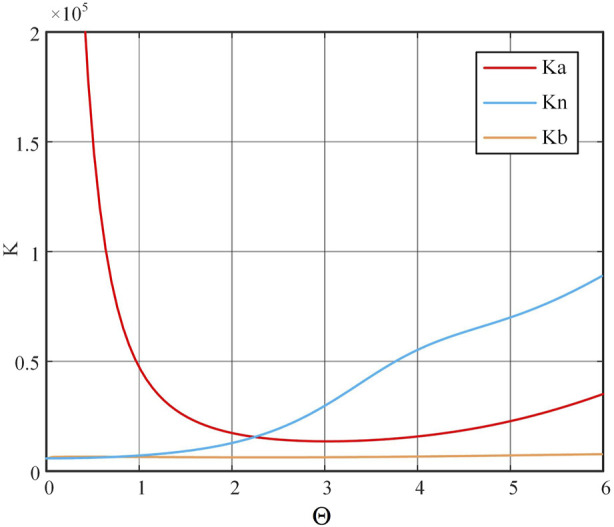
The continuum drive cable’s coupled model.

Taking the total bending angle as a variable, the computed results are depicted in Figure 8. It is evident that the trends align closely with those of an ideal continuum; as the bending angle increases, $K_{a}$ gradually decreases, $K_{n}$ exhibits increased fluctuations, and $K_{b}$ demonstrates the U shaped trend, albeit with minimal magnitude of variation.

Analyzing the stiffness model of the continuum and the cables, as concluded earlier, the deformation can be expressed as Equation 17. The stiffness model of the continuous system’s end under spatial loads can be represented by Equation 18.

### 3.4 Dual-section notched continuum robot stiffness model

**FIGURE 9:**
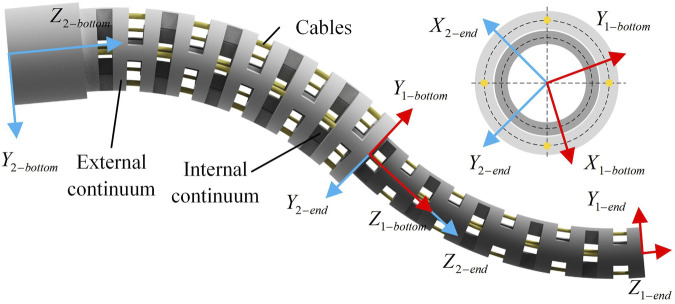
Schematic diagram of establishing the coordinate system of a dual-segment notched continuum robot.

As shown in Figure 9, the dual-segment configuration studied in this paper consists of nested outer and inner continuum with different diameters and lengths. Both inner and outer continuum are purely sectional. The geometric parameters of the single-segment continuous body are provided in Section 3.2. In this section, to distinguish between the inner and outer continuum, the inner tube is designated as Tube 2 and the outer tube as Tube 1. The following analysis considers the combined stiffness when both tubes are bent in arbitrary configurations. It is assumed that the bending planes of the two segments do not overlap, and the angle between these bending planes, denoted as γ , is between $Y_{1-bottom}$ and $Y_{2-end}$ .

Before proceeding with the subsequent derivations, this paper makes several assumptions to simplify the calculations regarding the model: 1). Frictional effects between the drive cable and the continuum are neglected. 2). The notches at the coupling interfaces of the two segments of the continuum overlap and have identical shapes. 3). The internal forces within both Segment two and Segment one at their junction interface are assumed to be exactly equal. 4). It is assumed that the bending directions of the two segments of the continuum lie within the same plane, and rotational effects of the continuum are disregarded. When the two segments of the continuum are coupling, the overall axial stiffness is the sum of the overall stiffness of the wire and the overall stiffness of the continuum.

$$K=K_{C}+K_{L}$$ (36)

In the setup, continuum 2 comprises a total of *i* segments of joints, while Continuum 1 comprises a total of 2*i* segments of joints. Aside from differences in length, diameter, and cross-sectional dimensions, all other parameters between Continuum 1 and Continuum 2 are identical. The overall bending angles at the coupled part of the continuum are represented as $\Theta_{1}$ , and the overall bending angles where the continuum is not involved in the coupling are denoted as $\Theta_{2}$ . $E_{\mathrm{continuum}}$ and $E_{\mathrm{cable}}$ refer to the elastic moduli of the continuum and the cable respectively, while GC and GL denote the shear moduli of the continuum and the driving cables. $IX_{\mathrm{C21}}$ , $IX_{\mathrm{C22}}$ , $IX_{C1}$ , $IY_{\mathrm{C21}}$ , $IY_{\mathrm{C22}}$ and $IY_{C1}$ respectively represent the moments of inertia of continuum two in the uncoupled part, continuum two in the coupled part, and continuum 1 in the X and *Y* directions.

After concatenation, the total energy of the continuum equals the sum of the total strain energies of continuum 1 and continuum 2.

$$V_{\varepsilon -C}=\overset{n}{\underset{m=1}{\sum }}{V_{\varepsilon }}^{m}+\overset{i}{\underset{m=1}{\sum }}{V_{\varepsilon }}^{m}$$ (37)

The total strain energy in the continuum section:

$$\begin{array}{c} V_{\varepsilon -C}=\overset{i}{\underset{1}{\sum }}V_{\varepsilon -C2}^{n}+\overset{i}{\underset{1}{\sum }}V_{\varepsilon -C1}^{n}\\ =\overset{i}{\underset{n=j}{\sum }}\left(\overset{l}{\underset{0}{\int }}\frac{{\left({F_{N}}^{n}\right)}^{2}}{2E_{C}A2}ds+\overset{l}{\underset{0}{\int }}\frac{k{\left({F_{s}}^{n}\right)}^{2}}{2G_{C}A2}ds+\overset{l}{\underset{0}{\int }}\frac{{\left(M^{n}\right)}^{2}}{2E_{C}I_{\mathrm{C21}}}ds\right)\\ \;+\overset{j}{\underset{n=1}{\sum }}\left(\overset{l}{\underset{0}{\int }}\frac{{\left({F_{N}}^{n}\right)}^{2}}{2E_{C}A2}ds+\overset{l}{\underset{0}{\int }}\frac{k{\left({F_{s}}^{n}\right)}^{2}}{2G_{C}A2}ds+\overset{l}{\underset{0}{\int }}\frac{{\left(M^{n}\right)}^{2}}{2E_{C}I_{\mathrm{C22}}}ds\right)\\ \;+\overset{m}{\underset{n=1}{\sum }}\left(\overset{l}{\underset{0}{\int }}\frac{{\left({F_{N}}^{n}\right)}^{2}}{2E_{C}A1}ds+\overset{l}{\underset{0}{\int }}\frac{k{\left({F_{s}}^{n}\right)}^{2}}{2G_{C}A1}ds+\overset{l}{\underset{0}{\int }}\frac{{\left(M^{n}\right)}^{2}}{2E_{C}I_{C1}}ds\right) \end{array}$$ (38)

where $F_{Nn}$ , $F_{Sn}$ , and $M_{n}$ are the stress conditions at the bottom of the *nth* joint of the continuum, with stress values derived recursively from the preceding section. *I* is the moment of inertia, and the expressions for different joints are distinct.

The strain energy of the cable is the sum of the total strain energy of the two segments of cable2 and the strain energy of cable1, namely,:

$$\begin{array}{c} V_{\varepsilon -L}=\overset{2}{\underset{1}{\sum }}V_{\varepsilon -L2}^{n}+V_{\varepsilon -L1}\\ =\left(\overset{l1}{\underset{0}{\int }}\frac{{\left({F_{N}}^{n}\right)}^{2}}{2E_{L}A}ds+\overset{l1}{\underset{0}{\int }}\frac{{\left(k{F_{s}}^{n}\right)}^{2}}{2G_{L}A}ds+\overset{l1}{\underset{0}{\int }}\frac{{\left(M^{n}\right)}^{2}}{2E_{L}I_{\mathrm{L21}}}ds\right)\\ \;+\left(\overset{l2}{\underset{l1}{\int }}\frac{{\left({F_{N}}^{n}\right)}^{2}}{2E_{L}A}ds+\overset{l2}{\underset{l1}{\int }}\frac{{\left(k{F_{s}}^{n}\right)}^{2}}{2G_{L}A}ds+\overset{l2}{\underset{l1}{\int }}\frac{{\left(M^{n}\right)}^{2}}{2E_{L}I_{\mathrm{L22}}}ds\right)\\ \;+\left(\overset{l2}{\underset{0}{\int }}\frac{{\left({F_{N}}^{n}\right)}^{2}}{2E_{L}A}ds+\overset{l2}{\underset{0}{\int }}\frac{{\left(k{F_{s}}^{n}\right)}^{2}}{2G_{L}A}ds+\overset{l2}{\underset{0}{\int }}\frac{{\left(M^{n}\right)}^{2}}{2E_{L}I_{L1}}ds\right) \end{array}$$ (39)

Given the known bending and deflection angles of the two-segment continuum, the stiffness of the end under axial force $F_{a}$ can be computed using the aforementioned method. Similarly, the total strain energy in the principal and secondary directions of the two-segment notched continuum can be calculated. Drawing an analogy to Equations 35–39, the stiffness models for the principal and secondary directions can be derived.

The stiffness model of the drive cable is established in Equation 40. When the continuum’s end is subjected to axial load $F_{a}$ , the stiffness of the drive screw can be calculated in two parts: uncoupled and coupled.

$$K_{a-L}=K_{a-LU}+K_{a-LUU}$$ (40)

where U is the coupled part, UU is the uncoupled part. The input force for the uncoupled part of the drive cable is simply $F_{a}$ , whereas for the coupled part, it should be the reaction force exerted on the lower end of the uncoupled part. Therefore, this study only needs to refer to Section 3.4 to separately calculate the stiffness of each part, and then linearly combine them. Similarly, when the continuum’s end is subjected to loads in the principal normal direction $F_{n}$ and secondary normal direction $F_{b}$ , the stiffness is defined in the same manner.

Compare the coupled stiffness in various directions between the continuum and the drive cable. Referring to Section 3.3, iterative calculations of the coupled stiffness models in each direction for the notched continuum and drive cable are conducted in MATLAB, with parameters provided in Section 2.

**FIGURE 10:**
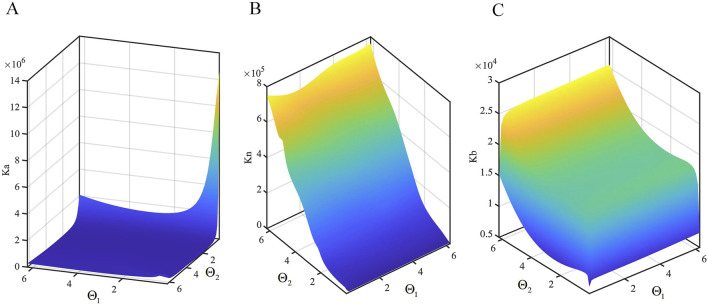
Variation of stiffness with angle. **(A)** Axial stiffness distribution of the continuum. **(B)** Principal normal stiffness distribution of the continuum. **(C)** Secondary normal stiffness distribution of the continuum.

Using the total bending angle $\Theta_{1}$ and of the two-segment continuum as variables, the three-dimensional graph of stiffness distribution is plot, as shown in Figure 10.

It can be observed that the axial stiffness $F_{a}$ of the continuum is greatly influenced by $\Theta_{1}$ and $\Theta_{2}$ from Figure 10A. When both angles approach zero, the axial stiffness reaches its maximum value, and the stiffness value sharply decreases as the angles in-crease. Furthermore, it is noted that for smaller values of θ , the stiffness curve shows significant variations, closely resembling the axial stiffness curve of a single-segment continuum. In contrast, for larger values of θ , the magnitude of stiffness variation diminishes, and the trend of change also alters., It can be seen that the principal normal stiffness $K_{n}$ is significantly influenced by $\Theta_{2}$ from Figure 10B. Specifically, as $\Theta_{1}$ increases, $K_{n}$ also increases, while the influence of $\Theta_{1}$ on is relatively small. When both $\Theta_{1}$ and $\Theta_{2}$ approach zero, $K_{n}$ reaches its minimum value, which closely resembles the trend of the principal normal stiffness of a single-segment continuum. It can be observed that the secondary normal stiffness $K_{b}$ is similarly greatly affected by $\Theta_{2}$ from Figure 10C, As $\Theta_{2}$ increases, $K_{b}$ initially increases rapidly, then its rate of in-crease slows down, and finally it increases rapidly again. Conversely, the impact on $K_{n}$ is relatively minor, and as $\Theta_{1}$ increases, $K_{b}$ initially increases rapidly followed by a slower rate of increase. Similarly, $K_{b}$ reaches its minimum value as $\Theta_{1}$ and $\Theta_{2}$ approach 0. This trend closely aligns with the behavior of secondary normal stiffness observed in a single continuum segment.

**FIGURE 11:**
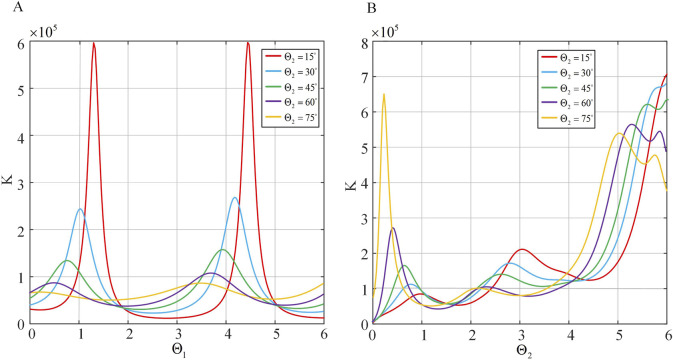
The stiffness in different angles. **(A)** Spatial stiffness distribution under different $\Theta_{1}$ conditions. **(B)** Spatial stiffness distribution under various $\Theta_{2}$ conditions.

Establish the spatial stiffness model for the two-segment continuum. From Section 3.3, let $\varphi_{a}$ , $\varphi_{n}$ , and $\varphi_{b}$ denote the angles between the spatial load *F* and the three force directions, respectively. The stiffness model of the two-segment continuum under the action of spatial loads can be expressed as Equation 18. To compare the stiffness under four different driving modes, the coupled stiffness under different conditions by varying the bending angles and angles of forces on the continuum are simulated and calculated in MATLAB. In the calculations, there are a total of five angle variables, denoted as $\Theta_{1}$ , $\Theta_{2}$ , $\varphi_{a}$ , $\varphi_{n}$ , and $\varphi_{b}$ . Since this study only considers the case where the bending angles of the two continua are in the same plane, $\varphi_{b}=90^{◦}$ . The simulation results are shown in Figure 11.

**FIGURE 12:**
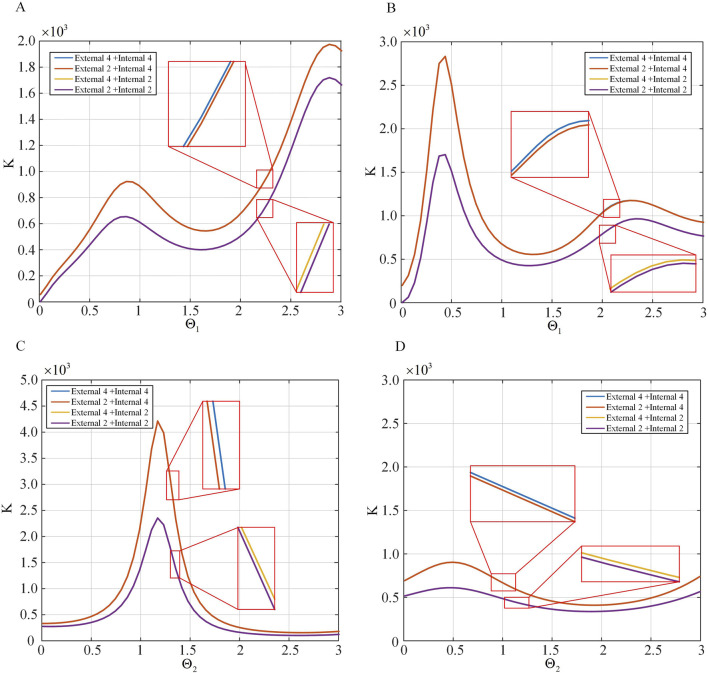
The spatial stiffness distribution for different numbers of driving strategies. **(A)** The spatial stiffness distribution under different $\Theta_{1}$ conditions. **(B)** The spatial stiffness distribution under various $\Theta_{1}$ conditions. **(C)** The spatial stiffness distribution under different $\Theta_{2}$ conditions. **(D)** The spatial stiffness distribution under various $\Theta_{2}$ conditions.

Comparison of spatial stiffness of the two-segment continuum under different driving strategies. The drive cable quantity is varied, thus changing the inertia moment and total cross-sectional area of the drive cables. Several sets of $\Theta_{1}$ and $\Theta_{2}$ are selected to compare the spatial stiffness of the continuum. Partial simulation results are shown in Figure 12, where Figures 12A, B vary with $\Theta_{1}$ , Figures 12C, D vary with $\Theta_{2}$ . From the figures, it can be observed that in practice, the stiffness of the continuum is nearly the same for the External 4+Internal 4 and External 2+Internal 4 strategies, and similarly for the External 4+Internal 2 and External 2+Internal 2 strategies. Overall, the relationship in magnitude is External 4+Internal 4 > External 2+Internal 4 > External 4+Internal 2 > External 2+Internal 2.

## 4 Experiment and result analysis

In this section, the continuum experimental platform is built and the basic performance is tested. The stiffness model verification experiment based on the experimental platform is carried out. Finally, the stiffness of the series continuum under different driving strategies is compared experimentally.

### 4.1 Experimental platform testing

**FIGURE 13:**
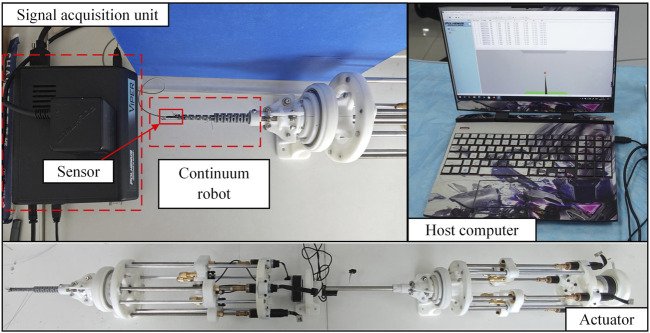
The prototype system of continuum robot experimental platform.

The serial continuum experimental platform constructed in this study is depicted as shown in Figure 13. Two segments of continuum robots are respectively mounted at the front end. Additionally, the bending angle of the continuum robot is measured using a single electromagnetic tracking sensor installed at the end-effector of the robot. The sensor provides real-time position and orientation data of the end-effector. The bending angle θ is calculated based on the direction vector D obtained from the sensor and the initial direction vector $D_{0}$ . The calculation formula is given by Equation 41.

$$\theta =\arccos\left(\frac{D\cdot D_{0}}{\left\|D\|\|D_{0}\right\|}\right)$$ (41)

where D is the direction vector measured by the sensor, and $D_{0}$ is the initial direction vector of the robot.

Motion tests on the inner and outer continuum bodies at various angles are conducted. Due to the material characteristics of the continuum robot, the bending angle between the two segments to be less than $60^{◦}$ during the experiments are controlled. This setup is sufficient for small deformation angles. For larger deformations, the use of an array of sensors may be considered in future work to enhance measurement accuracy.

### 4.2 Stiffness model verification

**FIGURE 14:**
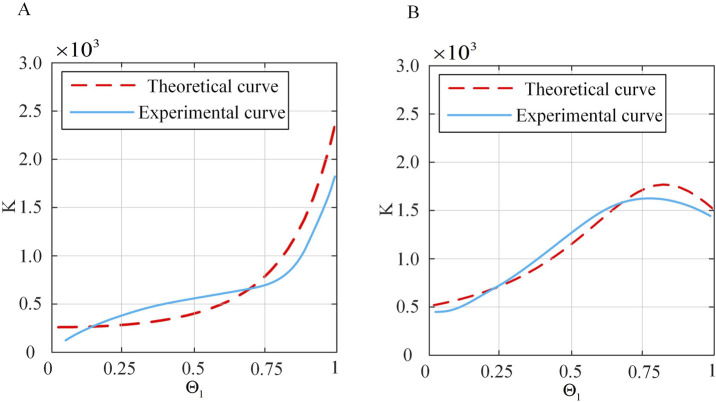
Comparison of theoretical and actual stiffness of the continuum under constant $\Theta_{1}$ . **(A)** $\Theta_{2}=20^{◦}$ . **(B)** $\Theta_{2}=40^{◦}$ .

**FIGURE 15:**
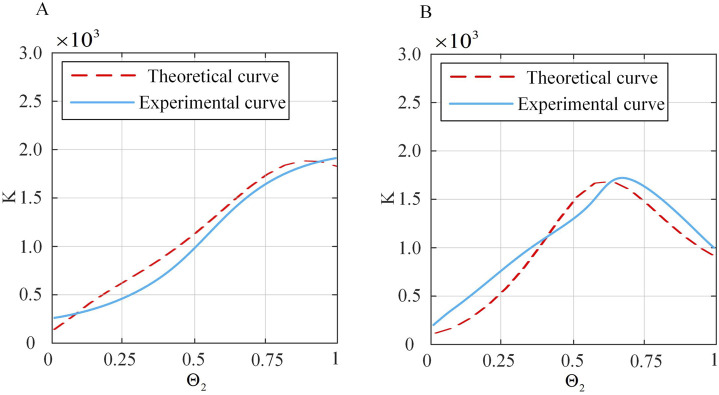
Comparison of theoretical and actual stiffness of the continuum under constant $\Theta_{2}$ . **(A)** $\Theta_{1}=15^{◦}$ . **(B)** $\Theta_{1}=35^{◦}$ .

To validate the spatial stiffness model proposed in Section 3.4 a series of experiments are designed in this paper. The continuum robot is bent to a certain extent, and electromagnetic tracking sensors are used to detect its initial position. The weight of 20 g is attached to the end of the continuum robot to provide a constant force, and the change in end position is monitored simultaneously. This allowed for the calculation of the overall stiffness of the continuum robot. Initially, $\Theta_{2}$ is kept constant while gradually increasing $\Theta_{1}$ . Displacement data for two sets of continuum robots are measured to obtain stiffness curves, as shown in Table 2 and Figure 14. Subsequently, $\Theta_{1}$ is kept constant while gradually increasing $\Theta_{2}$ and displacement data are collected to derive stiffness curves, as shown in Figure 15, with corresponding data shown in Table 3 and Figure 15. To ensure clarity and readability, the corresponding figures display the average values with error bars representing the standard deviations.

**TABLE 2:** Experimental data of stiffness with invariable $\Theta_{1}$ .

$\Theta_{2}{/}^{◦}$	$\Theta_{1}{/}^{◦}$	Displacement/mm	$\Theta_{2}{/}^{◦}$	$\Theta_{1}{/}^{◦}$	Displacement/mm
20	10	0.966	40	10	0.386
	25	0.337		25	0.239
	40	0.274		40	0.132
	50	0.261		50	0.191

**TABLE 3:** Experimental data of stiffness with invariable $\Theta_{2}$ .

$\Theta_{1}{/}^{◦}$	$\Theta_{2}{/}^{◦}$	Displacement/mm	$\Theta_{1}{/}^{◦}$	$\Theta_{2}{/}^{◦}$	Displacement/mm
15	10	0.623	35	10	0.166
	25	0.470		25	0.114
	40	0.393		40	0.078
	50	0.260		50	0.121

From Figures 14, 15, it can be seen that under small deformation angles, the actual stiffness of the continuum follows a trend closely aligned with the theoretical stiffness curve. This confirms that the spatial stiffness model proposed in Section 3.4 largely conforms to the practical model. However, there are still errors. Preliminary analysis shows that the sources of errors are as follows: (1) The influence of friction is not considered in the theoretical model. (2) The constant curvature model is used for continuum modeling, which has errors. (3) There are systematic errors and accidental errors in the experiment. (4) There are errors in the selection of actual material characteristic parameters, such as Young’s modulus, shear modulus and other parameters.

### 4.3 Continuum stiffness comparison

The verification work on the stiffness relationships of serial continuum under different driving strategies proposed in Section 3.4 is conducted. The experimental setup is identical to the previous subsection. Due to the conclusion that under the External 4+Internal four and External 2+Internal 4 strategies, the stiffness of continuum is nearly identical, as is the case under the External 4+Internal two and External 2+Internal 2 strategies, with an overall hierarchy of External 4+Internal 4 > External 2+Internal 4 > External 4+Internal 2 > External 2+Internal 2, serial continuum satisfy this regardless of angle. Thus, experiments are conducted with randomly assigned angle values for two-stage continuum, only changing the number of drive cables. The experimental results are shown in Table 4. Table 4 presents the experimental data for stiffness comparison under different driving strategies. The input variables are the driving strategies (e.g., External 4+Internal 4, External 2+Internal 4), while the output variables are the measured displacements under these strategies.

**TABLE 4:** Stiffness experimental data with fixed $\Theta_{1}$ .

Strategy	Displacement/mm	Order	Strategy	Displacement/mm	Order
External 4+Internal 4	0.102	1	External 4+Internal 4	0.083	1
External 2+Internal 4	0.110	2	External 2+Internal 4	0.086	2
External 4+Internal 2	0.147	3	External 4+Internal 2	0.101	3
External 2+Internal 2	0.171	4	External 2+Internal 2	0.106	4

It can be observed that the stiffness order under the four driving strategies is generally consistent with the theoretical results, but there are numerical errors that may cause the conclusion that under the External 4+Internal 4 and External 2+Internal 4 strategies, the stiffness of continuum is nearly identical, as is the case under the External 4+Internal 2 and External 2+Internal 2 strategies to be less obvious.

## 5 Conclusion

Basic performance evaluation of continuum robots is beneficial to their operation optimization and precise control in neurosurgery. The basic performance of continua is mainly evaluated by stiffness, but there is no systematic and universal evaluation system. In order to realize the driving and testing of continuum with different configurations and different driving strategies, this paper designs a universal experimental platform for continuum robot, and the continuum stiffness evaluation method based on the energy method and Castigliano’s second theorem is proposed. By solving the internal force and energy of the cut continuum in sections, the stiffness model of single-segment and double-segment series continuum is established. The relationship between the individual stiffness and bending angle of the single-segment continuum and the driving cables in the axial direction, main normal direction, and secondary normal direction is obtained. The simulation and experimental results show that under the condition of small deformation angle, the spatial stiffness model obtained by strain energy basically conforms to the actual model, which verifies the correctness and rationality of the stiffness calculation method proposed in this paper. Future work will explore the incorporation of nonlinear material properties and geometric nonlinearities to extend the model’s applicability to larger deformations.

## Data Availability

The original contributions presented in the study are included in the article/supplementary material, further inquiries can be directed to the corresponding authors.
